# Omega-3 and Omega-6 Polyunsaturated Fatty Acid Intakes, Determinants and Dietary Sources in the Spanish Population: Findings from the ANIBES Study

**DOI:** 10.3390/nu15030562

**Published:** 2023-01-21

**Authors:** Marina Redruello-Requejo, María de Lourdes Samaniego-Vaesken, Ana M. Puga, Ana Montero-Bravo, Mar Ruperto, Paula Rodríguez-Alonso, Teresa Partearroyo, Gregorio Varela-Moreiras

**Affiliations:** 1 Grupo USP-CEU de Excelencia “Nutrición Para la Vida (Nutrition for Life)”, Ref: E02/0720, Departamento de Ciencias Farmacéuticas y de la Salud, Facultad de Farmacia, Universidad San Pablo-CEU, CEU Universities, 28660 Boadilla del Monte, Spain; 2 Spanish Nutrition Foundation (FEN), c/General Álvarez de Castro 20, 1 apta, 28010 Madrid, Spain

**Keywords:** ANIBES study, Spanish population, nutrition assessment, dietary habits, food sources, omega-3, omega-6, EPA, DHA, folic acid, vitamin B_12_, choline

## Abstract

The multiple roles of polyunsaturated fatty acids (PUFA) in growth and general health are well documented. However, available intake data for the Spanish population are limited and lack gender and age considerations. Therefore, our goal was to assess dietary intake adequacy of omega-3 and omega-6 PUFA, their determinants and their major food sources among the Spanish population. Due to their influence on various beneficial functions attributed to omega-3 PUFA, combined intake adequacy with folic acid (FA), vitamin B₁₂ and choline was also assessed. Intake data were obtained from the ANIBES cross-sectional study on a representative sample of the Spanish population (9–75 years; *n* = 2009), where dietary intake was analysed with a three-day dietary record. Median intake of total omega-3 PUFA stood at 0.81 g/day (0.56–1.19 g/day), with α-linolenic acid (ALA) at 0.61 g/day (0.45–0.85 g/day), eicosapentaenoic acid (EPA) at 0.03 g/day (0.01–0.12 g/day) and docosahexaenoic acid (DHA) at 0.06 g/day (0.0–0.20 g/day). Accordingly, 65% of the Spanish population showed insufficient intakes for total omega-3 PUFA; 87% for ALA, and 83% for combined EPA and DHA. Inadequate intakes were significantly higher in children, adolescents, and younger women of childbearing age (18–30 years). In contrast, inadequacy due to excessive intakes was almost negligible. Regarding omega-6 PUFA, total intake was 10.1 g/day (7.0–14.0 g/day), 10.0 g/day (6.9–13.9 g/day) for linoleic acid (LA) and 0.08 g/day (0.05–0.13 g/day) for arachidonic acid (AA). Non-compliance due to either insufficient or excessive intakes of LA stood at around 5% of the sample, with the elderly showing significantly higher degrees of inadequacy due to insufficient intakes (10%; *p* ≤ 0.05). Median omega-6 to omega-3 ratio was 12:1, and significantly higher in men compared to women (*p* ≤ 0.05); in children, adolescents and adults compared to the elderly (*p* ≤ 0.05); and in younger women of childbearing age compared to the older group (31–45 years) (*p* ≤ 0.001). Oils and fats and meat and meat products were the main dietary sources for the essential fatty acids LA and ALA, respectively. Meat and meat products were as well the main providers of AA, while fish and shellfish were almost exclusively the only sources of EPA and DHA. However, main food sources identified showed important differences across age groups. Finally, the total combined degree of inadequacy observed for omega-3 PUFA, FA, vitamin B₁₂ and choline reached 21.3% of the ANIBES population. The observed degree of inadequacy of omega-3 PUFA intakes among the Spanish population makes it urgent to increase its consumption and to consider the need for supplementation. This should also be the main strategy for the optimization of the omega-6/omega-3 ratio, as the adequacy observed for omega-6 intakes is relatively acceptable. Additional improvement of the dietary intake of FA, vitamin B_12_ and choline could contribute to the beneficial effects of omega-3 PUFA.

## 1. Introduction

### 1.1. Metabolism of Omega-6 and Omega-3 Polyunsaturated Fatty Acids

Docosahexaenoic acid (DHA) has received significant attention in the last two decades due to its key role in health and disease prevention. DHA is a long-chain polyunsaturated fatty acid (LCPUFA) belonging to the omega-3 (ω3) series of polyunsaturated fatty acids (PUFA), alongside eicosapentaenoic acid (EPA) and docosapentaenoic acid (DPA). These can be directly consumed in the diet or synthesized from α-linolenic acid (ALA), the essential omega-3 PUFA, as it cannot be synthesized in the body and thus must be obtained through the diet. Along with the omega-3 series, PUFA also include the omega-6 (ω6) series of fatty acids, which comprises, among others, another essential fatty acid, linoleic acid (LA), and its main LCPUFA product, arachidonic acid (AA), which can be obtained through the diet.

**Figure 1:**
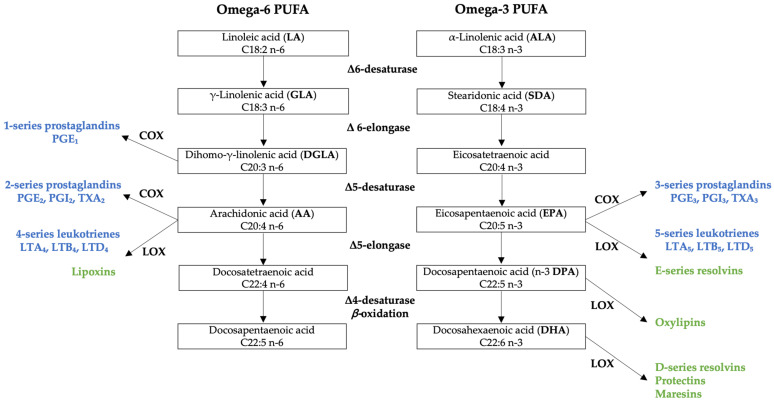
Metabolic routes undergone by omega-6 and omega-3 polyunsaturated fatty acids (PUFA) as precursors of eicosanoids (in blue) and specialized pro-resolving lipid mediators (SPMs, in green). COX: cyclooxygenases; LOX: lipoxygenases.

PUFA have special relevance in overall health status due to the particularity of giving rise to certain biologically active lipid mediators that act as potent intracellular regulators, playing key roles in inflammatory processes and in immune responses [1]. However, for these processes to take place properly, a suitable balance between omega-3 and omega-6 PUFA intake is a key factor because both compete in the metabolic pathways involved in the synthesis of those bioactive lipid mediators [2] (Figure 1).

The two essential fatty acids, LA, and ALA, give rise to their respective LCPUFA undergoing a similar metabolic route, in which desaturation and elongation enzymes are shared. Although LCPUFA can also be consumed in the diet, the ultimate transformations leading to the formation of active lipid mediators will depend on enzymatic competition, because in addition to the aforementioned enzymes, both series also further compete for cyclooxygenases (COX) and lipoxygenases (LOX) leading to the formation of eicosanoids (prostaglandins and leukotrienes) [2]. Specifically, omega-6 AA will give rise to potent inflammatory and pro-aggregatory eicosanoids termed 2-series prostaglandins and 4-series leukotrienes [3]. And although omega-6 PUFA have traditionally been regarded to as pro-inflammatory compounds, dihomo-γ-linolenic acid (DGLA), another omega-6 LCPUFA, is the precursor of 1-series prostaglandins, which have been shown to have anti-inflammatory, vasodilative and anti-hypertensive effects [4]. From the omega-3 series, specifically from EPA, derive 3-series prostaglandins and 5-series leukotrienes with anti-inflammatory, anti-aggregatory and vasodilative functions [3]. LOX enzymes will also enable the synthesis of the so-called specialized pro-resolving lipid mediators (SPMs), which further help extinguish the inflammatory process [5]. The most well-known SPMs derive from the omega-3 series of LCPUFA, such as E-series resolvins, formed from EPA; D-series resolvins, protectins and maresins, forming from DHA; and oxylipins, from DPA. Omega-6 series are also involved in the resolution of inflammation, as other SPMs called lipoxins are formed from AA [3].

The shared metabolic routes of the two series illustrate the functional connections of the precursors and the products involved. Given that both series depend on enzymatic competition, the cell membrane concentration of its precursors will determine the relative levels of the products formed. It is well established that Western-type diets tend to include insufficient omega-3, while omega-6 intake tends to be higher than recommended, and as a result cell membranes usually contain higher amounts of AA [2]. Thus, the strategy to follow should aim to promote the presence and metabolism of the 3-series by optimizing the composition of dietary fatty acids, so that providing more EPA and DHA could ultimately modulate eicosanoid formation and reduce the state of chronic low-grade inflammation associated to a growing array of chronic diseases [2]. Indeed, healthier dietary patterns, as measured by the Healthy Eating Index (HEI), are associated with lower omega-6/omega-3 ratios [6] and adherence to the Mediterranean diet has a correlation with red blood cell membrane concentrations of AA, EPA and DHA [7].

Under this strategy, it is important to emphasize the very limited metabolic conversion of dietary ALA into EPA and especially into DHA, which may be insufficient to support optimal health in significant portions of the population, specifically those excluding fish and shellfish from their diets [8]. Interestingly, the conversion rate into DHA is greater in females and is further up-regulated during pregnancy, presumably due to hormonal factors. Nonetheless, conversion rates would still be minimal, with supplementation of pregnant women with ALA failing to provide increased umbilical blood levels of DHA [9]. In contrast, supplementation of pregnant women with fish oil, rich in DHA, increased umbilical blood levels and infant plasma levels of DHA at birth [10]. Likewise, maternal dietary DHA has a strong dose-dependent effect on DHA concentration in breast milk [11].

### 1.2. Biological Functions and Adequate Intakes of Omega-6 and Omega-3 Polyunsaturated Fatty Acids

PUFA are constituents of cell membranes, significantly determining its fluidity and function, and within plasma lipoprotein particles, PUFA are also major constituents of phospholipids, triglycerides, and cholesterol esters. In addition, they serve as precursors to bioactive metabolites that will act as secondary messengers and mediate functions such as inflammatory responses, platelet aggregation and vascular tone [12].

As previously stated, among PUFA, omega-6 LA and omega-3 ALA are the only essential fatty acids, because these are nutrients that must be consumed as such in the diet due to our inability to synthesize them. From a physiological perspective, LA and ALA are also essential nutrients required for growth, development, and normal functioning. This is proven by the effects of its deficiencies, which constitute a pathological entity known as essential fatty acid deficiency (EFAD). Manifestations of EFAD include hyperlipidemia, thrombocytopenia, altered platelet aggregation and elevated hepatic enzymes, as well as other clinical signs like increased susceptibility to infection, reduced growth in infants and children, impaired wound healing, dry scaly rash, or hair loss [12]. EFAD is relatively rare, as sufficient intakes to prevent this situation are estimated to be 2%–4% of total energy intake for LA and 0.25%–0.5% for ALA, but can occur in individuals severely limiting fat intake [13].

In addition, the limited and widely-variant conversion rate of ALA into DHA has led to growing recognition of this nutrient as a conditional essential fatty acid [14]. Indeed, DHA plays a crucial role in several life stages:

In infants DHA is deemed critical for the growth and functional development of the brain, as well as for the maintenance of normal brain function in adults [15]. Brain tissues (mainly the grey matter) and eye contain higher proportions of DHA compared to other organs [16], as it is involved in neuronal signaling and in visual function [17]. While maintenance of DHA concentration is vital during the whole life course, particularly pregnancy, lactation and infancy are especially vulnerable periods, in which the role of DHA in the first 1000 days of life is therefore of major importance for subsequent optimal fetal development and ultimately adequate intakes of the newborn [13]. In this regard, studies show that supplementation with EPA and/or DHA may reduce pregnancy complications and benefit different maternal and newborn outcomes, with an anti-inflammatory effect on the placenta [18,19,20]. Furthermore, positive associations between maternal omega-3 PUFA intake during early pregnancy and child neuropsychological functions were found in the study by Tahaei et al. [21], with prospective data from 2644 pregnant Spanish women. Compared to participants in the lowest quartile (<1.262 g/day) of omega-3 PUFA consumption during the first trimester, those children whose mothers were in the highest quartile (>1.657 g/day) had a higher general cognitive score.

In addition, the fatty acid profile of the diet is a known determinant of cardiovascular risk, where decreasing the contribution of saturated fatty acids and total fat, while increasing the intake of PUFA, as in fish oil, helps lower this risk [1]. The meta-analysis performed by He et al. [22] on cohort studies indicated that each daily increase of 20 g in fish intake was associated to a 7% lower risk of mortality due to coronary heart disease (CHD). Comparable conclusions were obtained for stroke being the endpoint. Another recent meta-analysis by Harris et al. [23] showed a continuous significant dose response relationship for the combined intake of EPA and DHA (up to 0.5 g/day) and the risk of CHD-related death.

Finally, existing evidence from cross-sectional and prospective observational studies point to an inverse association between dietary and supplemental intake of omega-3 PUFA and the risk of cognitive decline, dementia, and Alzheimer’s disease [24,25]. Although the available evidence to date is controversial and not conclusive, several systematic reviews on observational studies and clinical trials would confirm the positive effect of omega-3 fatty acids -particularly DHA- on cognition, mainly in memory functions, of older adults and the elderly [26,27]. Anyway, additional long-term intervention clinical trials are needed to assess the role of omega-3 fatty acids and to ascertain which dosage and duration of supplementation should be more efficient.

The nutritional goals established by the Spanish Society of Community Nutrition (SENC) regarding total PUFA are set at 4% of total energy (TE), particularly 1–2% TE for omega-3 fatty acids, including 0.2 g/day of DHA and the combination of EPA + DHA at 0.5–1 g/day. The proposed figures are adapted to the fish consumption habits of the Spanish population, considering an average fish intake of one portion per week and 2 portions per week for the 75th percentile [28]. However, the SENC does not detail recommendations for specific population groups, as does the European Food Safety Authority (EFSA). For pregnant and nursing women, the Adequate Intake (AI) adds 0.1–0.2 g/day of preformed DHA to that of adults, set at 0.25 g/day for EPA + DHA [1]. For infants, an AI of 0.1 g/day of DHA is proposed for children aged >6 months to 24 months, as intake levels between 0.05–0.1 g/day have been found effective for visual function during the complementary feeding period. For the age period 2 to 18 years, the EFSA only states that dietary recommendations for children should be consistent with those of adults [1]. In the present work, in order to compare the results obtained with the available evidence we considered the recommendations established by the Food and Agriculture Organization of the United Nations/World Health Organization (FAO/WHO) in 2008 [13], where PUFA contribution should range between 6–11% TE, with omega-3 fatty acids between 0.5–2% TE and EPA + DHA at 0.25–2 g/day. For adult pregnant and lactating females, the minimum intake aiming for optimal adult health and fetal and infant development is set at 0.3 g/day EPA + DHA, with at least 0.2 g/day of DHA [13].

Although the multiple roles of polyunsaturated fatty acids (PUFA) in growth and general health are well documented, available intake data for the Spanish population are limited and lack gender and age considerations. One of the main studies assessing omega-3 dietary sources and intake adequacy was conducted by Ortega et al. [29] on a representative sample of Spanish adults (*n* = 1068 adults, 521 men and 547 women, aged 17–60 years, from ten Spanish provinces). Intake was assessed in year 2008 with a food record on 3 consecutive days, including a Sunday. Results showed inadequate PUFA contribution, since 79.2% of the subjects had an intake <6% of TE. Particularly, contribution of omega-3 PUFA (1.85 ± 0.82 g/day) provided less than 1% of TE in 85.3% of subjects and the combination of EPA + DHA (0.55 ± 0.58 g/day) did not exceed 0.5 g/day in 64.6% of the sample. Interestingly, the ECLIPSES longitudinal Study [30] on 479 Spanish pregnant women reported that those of older age and higher educational level showed significantly higher EPA and DHA serum concentrations and lower values of the omega-6/omega-3 and AA/EPA ratios.

In the present study we decided to jointly evaluate the dietary intake adequacy of omega-3 PUFA along with some methionine and methylation-cycle related vitamins and cofactors, namely folic acid (FA), vitamin B₁₂ and choline. Another work from the ANIBES Study carried out in Spanish women of childbearing age [31] has already demonstrated the need to optimize the dietary intake of these micronutrients that share many functions with those of omega-3 PUFA. Because of their implication in one-carbon metabolism, FA, vitamin B₁₂ and choline play a key role in the regulation of plasmatic homocysteine, with elevated levels being associated to several negative health outcomes such as cerebrovascular events, ischemic heart disease, cognitive impairment and dementia [32]. As previously stated, the nutritional status in omega-3 (and omega-6) PUFA may also have a direct influence in such outcomes. In addition, mounting evidence suggests choline to be a critical perinatal nutrient with functions that may potentially overlap with those of DHA [33,34].

For all the aforementioned, our objective was to analyze and evaluate dietary intake adequacy of omega-3 and omega-6 PUFA and their major food sources amongst the Spanish population from the ANIBES study sample. In addition, the combined dietary intake adequacy of omega-3 PUFA with FA, vitamin B₁₂ and choline has been analyzed.

## 2. Materials and Methods

### 2.1. Study Design and Sample

The protocol and methodology of the ANIBES study has been previously described in detail [35]. Briefly, it is a cross-sectional study with stratified multistage sampling whose fieldwork was performed from mid-September 2013 to mid-November 2013 at 128 sampling points across Spain. The ANIBES study aimed to comprise a sample size that is representative of all individuals living in Spain, aged 9–75 years, and living in municipalities of at least 2000 inhabitants. The final sample included 2009 individuals (1013 men, 50.4%; 996 women, 49.6%). Additionally, for the youngest age groups (9–12, 13–17, and 18–24 years), an “augment sample” was included to comprise at least *n* = 200 per age group (error ± 6.9%). Therefore, the total final sample included 2285 participants. Sample quotas were considered for age groups (9–12, 13–17, 18–64, and 65–75 years), gender (men/women), geographical distribution (Northeast, East, Southwest, North–Central, Barcelona, Madrid, and Balearic and Canary Islands) and locality size: 2000 to 30,000 inhabitants (rural population); 30,000 to 200,000 inhabitants (semi-urban population), and over 200,000 inhabitants (urban population). In addition, women of childbearing age (18–45 years, *n* = 552) were divided into two groups: younger women of childbearing age (18–30 years, *n* = 209) and older women of childbearing age (31–45 years, *n* = 343). The following exclusion criteria was applied: individuals living or staying at colleges, hospitals, and other institutions; individuals working in fields related to market research, advertising, or journalism; individuals with acute diseases (common cold, gastroenteritis, chickenpox, etc.), with allergies or intolerances, and those with metabolic diseases such as hyperthyroidism or hypothyroidism. In addition, individuals strictly following a specific diet were also excluded; as well as pregnant or lactating women and individuals following recommendations for diabetes, hypertension, hypercholesterolemia, hypertriglyceridemia, or hyperuricemia. Finally, other factors were considered for sample adjustment: unemployment rate, percentage of immigrant population, tobacco use, and educational and economic level. The final protocol was approved by the Ethical Committee for Clinical Research of the Region of Madrid, Spain [36].

### 2.2. Dietary Survey and Data Collection

Participants were given a tablet device (Samsung Galaxy Tab 2 7.0, Samsung Electronics, Suwon, Korea) and were trained to record information and take photographs of all food and drinks consumed, both at home and outside, throughout the three days of the study (two weekdays and one weekend day). Actual food and beverage intake was assessed with photographs of each initial and final eating or drinking event, to account for leftovers. Additionally, participants also recorded a brief description of meals, recipes, and brands, along with the specific situation in which they ate, whether they were eating with others, watching television, or sitting at the table. Dietary supplement use was also recorded. For each day, participants were asked to indicate whether that day’s intake was representative of their normal life, or the reasons why it was not. Food records were returned from the field in real time, and an ad hoc central server software/database was developed for this purpose, to work in parallel with the codification and verification processes.

Energy and nutrient intakes were estimated from food consumption records using the VD-FEN 2.1 software, a Dietary Evaluation Program developed by the Spanish Nutrition Foundation (FEN), Spain, based mainly on Spanish food composition tables [37], with several expansions and updates. For choline, which is currently not included in European food composition databases, we referred to the USDA National Nutrient Database for Standard Reference [38] and to the USDA Database for the Choline Content of Common Foods [39].

Omega-3 and omega-6 PUFA intake adequacy was evaluated with respect to the Food and Agriculture Organization of the United Nations/World Health Organization (FAO/WHO) dietary recommendations [13], while intake adequacy of FA, vitamin B₁₂ and choline was evaluated according to the EFSA dietary reference values [40].

### 2.3. Anthropometric Measurements

In addition, several anthropometric measurements were collected by trained interviewers following the procedures previously tested at two pilot studies. Height was measured by triplicate using a Stadiometer model Seca 206 (Seca, Hamburg, Germany) and weight was determined with one measurement in a weighing scale model Seca 804 (Seca, Hamburg, Germany).

### 2.4. Statistical Analysis

Kolmogorov-Smirnoff normality test was performed to determine the normality of the distribution of the variables. Median and interquartile range (IQR) were used for continuous variables and frequencies and percentages for categorical variables. Kruskal–Wallis and Dunn tests were used to adjust for multiple comparison. *p*-value was adjusted with Bonferroni’s correction to calculate differences among each age group within samples. Mann–Whitney U test correction was used to calculate differences by gender. Level of significance was established at *p* ≤ 0.05. Data analyses were performed with IBM SPSS 27.0 (IBM Corp., Armonk, NY, USA).

## 3. Results

### 3.1. Omega-3 and Omega-6 PUFA Dietary Intakes

Median daily intakes of total omega-3 fatty acids, ALA, EPA, and DHA for the Spanish population are shown in Table 1. Within the total population, male participants had significantly higher total omega-3 and ALA intakes than females (*p* ≤ 0.001). Within age-groups, no significant differences were observed regarding total omega-3 and ALA intakes. However, significant differences (*p* ≤ 0.05) were observed amongst adolescent and adult EPA and DHA intakes and, thus, in the sum of DHA and EPA when compared to the elderly.

Table 2 shows the median daily intakes of total omega-3 fatty acids, ALA, EPA, and DHA observed for women of childbearing age. Intake levels were similar to those of the group of adult women included in the ANIBES population. However, significantly lower intakes were observed for younger women of childbearing age when compared to the older group regarding total omega-3 PUFA (*p* ≤ 0.05), EPA (*p* ≤ 0.001), DHA (*p* ≤ 0.01) as well as for combined EPA + DHA (*p* ≤ 0.01).

Table 3 shows total omega-6 fatty acids, LA, and AA median intakes in the entire population and different gender and age groups from the ANIBES study. Male participants had significantly higher omega-6, LA, and AA intakes than females (*p* ≤ 0.001). When results were analysed by age-groups, we observed that total omega-6, LA, and AA intakes were significantly lower with advancing age (*p* ≤ 0.05). Specifically, higher reported intakes were observed in children and adolescent groups compared with adults and elderly groups (*p* ≤ 0.05).

Concerning women of childbearing age, no significant differences were found in the consumption of any of the omega-6 fatty acids (Table 4).

Table 5 displays the omega-6/omega-3 and EPA/DHA intake ratios for the ANIBES Spanish population, segmented by gender and age. No significant differences were observed regarding EPA/DHA ratio in the entire population. However, we observed the omega-6/omega-3 ratio to be significantly higher (*p* ≤ 0.05) in men compared to women and in children, adolescents and adults when compared to that of the elderly.

Regarding women of childbearing age, we observed (Table 6) a significantly greater omega-6/omega-3 intake ratio for those of younger age (*p* ≤ 0.001) when compared to the older group. Again, no significant differences were found for the EPA/DHA ratio.

Furthermore, the total sample was segmented by body weight into four groups (underweight, normal weight, overweight and obese) according to BMI recommended by WHO for children [41] and adults [42]. As shown in Table 7, individuals with obesity showed significantly lower omega-6 fatty acid intakes and omega-6/omega-3 ratio than those underweight. However, no significant differences were observed related to omega-3 fatty acid intakes nor to the EPA/DHA ratio.

When segmented by geographical areas of residence (Table 8), we found no differences in omega-3 and omega-6 fatty acid dietary intake and ratios across Spain.

Table 9 shows the degree of non-compliance with the nutritional goals for PUFA established by the FAO/WHO [13]. In general, for omega-3 fatty acids a high degree of non-compliance is observed, with more than half of the population showing intakes below recommendations and no significant differences found with respect to gender. Children and adolescents showed significantly higher degrees of non-compliance for total omega-3 than adults and the elderly. In fact, 78% of children and adolescents fail to reach 0.5% of TE from omega-3 fatty acids. Regarding the nutritional goals for ALA, all age groups showed a non-compliance of more than 80%, with inadequacy being again significantly higher amongst the younger age groups when compared to adults and elders. When assessing non-compliance with the nutritional objectives for EPA + DHA, it was found that, once again, more than 80% of individuals had intakes <0.25 g/day, the proportion being significantly higher for adolescents. In contrast, the degree of non-compliance due to intakes above recommendations for omega-3 fatty acids was almost negligible. Regarding omega-6 fatty acids, population failing to meet the nutritional goals for LA was not remarkable, and again no significant differences were found between genders. Inadequate intakes comprised around 5% of the sample both for insufficient and excessive intakes, although the elderly (65–75 years) showed a significantly higher degree of non-compliance due to insufficient intakes than the rest of the population.

The degree of non-compliance with the nutritional goals for PUFA observed for women of childbearing age is shown in Table 10. Significant differences were only observed for total omega-3 PUFA intakes, where younger women of childbearing age had a significantly (*p* ≤ 0.05) higher degree of non-compliance with the nutritional goals, approximately 10% more than older women of childbearing age.

### 3.2. Omega-3 and Omega-6 PUFA Dietary Sources

To provide a general overview, Table 11 shows the dietary food sources of lipids observed for the ANIBES Study population, sorted by their contribution to total PUFA intake. Oils and fats were the main sources of PUFA intake among the ANIBES population, followed by meat and meat products, cereals and derivatives, and fish and shellfish.

The dietary sources of omega-3 PUFA (total, ALA, EPA, and DHA) are detailed in Table 12. Meat and meat products and fish and shellfish and were the main sources of total omega-3 PUFA, contributing to similar amounts. However, fish and shellfish were almost exclusively the only sources of DHA and EPA, while meat and meat products only provided ALA. Similarly, other important omega-3 sources were oils and fats and cereals and derivatives, which again only contributed with ALA.

When analyzing the dietary sources of total omega-3 intakes according to age groups (Table 13), meat and meat products were found to be the main source for children and adolescents, for which the observed contribution of this food group was significantly higher than that of the adult and the elderly populations (*p* ≤ 0.05). For adults, meat and meat products and fish and shellfish contributed in a similar way; and only in the elderly were fish and shellfish the main contributors to total omega-3 intakes. Notably, the contribution of fish and shellfish observed for children and adolescents was significantly lower compared to the older age groups (*p* ≤ 0.05). Only those foods that contributed at least 1% to total omega-3 intakes of the population have been included.

As already shown in Table 12, the main sources of ALA intakes among the ANIBES study population were meat and meat products, followed by oils and fats, cereals and derivates and milk and dairy products. This order of contribution remained across age groups (Table 14), except for the case of adolescents, where cereals and derivatives contributed slightly more to ALA intake than oils and fats. In contrast, the elderly showed a significantly higher contribution of this later food group to their ALA intakes (*p* ≤ 0.05).

Regarding EPA intake, fish and shellfish were almost exclusively the only food source in the whole sample population (Table 12) but contributed significantly more to the intakes of adults and elders (*p* ≤ 0.05) (Table 15). The contribution observed for younger groups was significantly lower, particularly in the case of adolescents, for which fish and shellfish and milk and dairy products provided similar amounts of EPA. In the other age groups, milk and dairy products contributed to similar amounts as in adolescents, but in a lesser extent relative to fish and shellfish.

Likewise, fish and shellfish represented the only dietary source of DHA (Table 16), showing a significantly higher contribution for the elderly (*p* ≤ 0.05).

Table 17 shows the contribution of dietary food sources for omega-6 PUFA (total, LA, and AA). Observed intakes of total omega-6 PUFA, mainly in the form of LA, were firstly provided by oils and fats, followed by meat and meat products and by cereals and derivatives. Regarding AA, only meat and meat products showed a relevant contribution.

The contribution of dietary food sources to total omega-6 PUFA among age groups is shown in Table 18. The order of contribution of the main food sources remained as described for the total population (Table 17), however, the contribution of cereals and derivates, ready-to-eat meals and milk and dairy products to omega-6 fatty acids intake was much higher among children or adolescents than among young and older adults (*p ≤* 0.05).

Observed intakes of total omega-6 PUFA were predominantly in the form of LA, thus the main food sources are common to both (Table 17) and again their order of contribution remained the same across age groups (Table 19). The same significant differences were also observed according to age for cereals and derivates, ready-to-eat meals and milk and dairy products (higher contributions in the youngest groups vs. older groups; *p* ≤ 0.05). In addition, the contribution of sauces and condiments to LA intake was significantly lower among the elderly than among the other age groups (*p* ≤ 0.05).

As for AA intake, meat and meat products were the greatest contributors across all age groups, followed by milk and dairy products and fish and shellfish (Table 20). Particularly, we observed that in children, adolescents and adults, meat and meat products had a significantly higher contribution to AA intakes than in the elderly (*p* ≤ 0.05). In addition, milk and dairy products showed a significantly higher contribution to AA intakes of children when compared to that of older adults (*p* ≤ 0.05).

### 3.3. Combined Dietary Intake Adequacy of Omega-3 PUFA, Folic Acid (FA), Vitamin B_12_ and Choline

Considering the nutritional goals set by the EFSA [40], 56.9% of the ANIBES population showed insufficient dietary intakes for both omega-3 PUFA and FA, with adolescents showing the lowest combined compliance rates. However, the elderly achieved the highest degree of compliance with these two nutritional goals, with inadequacy dropping to 48.5% of this population group. With respect to both omega-3 PUFA and vitamin B₁₂, the observed degree of non-compliance with the nutritional goals reached 23.1% of the population, and adolescents were as well the group with the highest non-compliance rates (26.5%). Regarding omega-3 PUFA and choline, 27.2% of the population showed combined inadequate intakes. Finally, the total combined degree of inadequacy observed for the nutrients considered (omega-3 PUFA, FA, vitamin B₁₂ and choline) reached 21.3% of the ANIBES population. More specifically, the population group with the greatest dietary insufficiency in these nutrients were those over 65 years of age (23.9%), followed by adolescents (22.7%), adults (21.9%) and, lastly, children with only 6.6%.

## 4. Discussion

### 4.1. Omega-3 and Omega-6 PUFA Dietary Intakes

Scientific evidence has made clear that not only the quantity but also the quality of dietary fats critically influences health [13]. Therefore, the present analysis focused on PUFA intake is of great relevance. In general terms, Western-type dietary patterns provide mainly LA (omega-6), a lower proportion of ALA (omega-3) and, depending on seafood intake, a variable but relatively low proportion of omega-3 LCPUFA, namely AA, EPA, DHA and DPA [13]. The previously mentioned study by Ortega et al. [29] in a Spanish sample already revealed an insufficient contribution of PUFA, specifically that of omega-3. Results obtained in the present work derived from the ANIBES Study [35] showed even more insufficient intakes. It is worth underlining that the fieldwork for the work by Ortega et al. was carried out in 2008 while the ANIBES study took place five years later, including a sample size of almost twice the number of participants. Furthermore, the study by Ortega and colleagues reported mean intake data, and not median as we have opted to do in the present work, in the light of other studies reporting that omega-3 intake, specifically that of EPA and DHA, showed great inter-individual variability and little homogeneity within the same population [43,44].

With regard to omega-3 fatty acids, in the present study we observed intakes of less than half of those reported by Ortega et al. [29]. Furthermore, it is worth noting that we observed significantly higher intakes for total omega-3 and ALA in men. The results obtained here are in line with other studies available in the European population. In French adults [43], with intake data from 1998, median intakes of ALA also did not exceed 1 g/day, while EPA and DHA intakes were higher and stood at 0.1 g/day and 0.2 g/day, respectively. Interestingly, no significant differences in consumption by gender were found. In Belgian women of childbearing age [44], with intake data from 2002, median intakes of ALA were 1.3 g/day, while EPA and DHA intakes were lower, at 0.01 g/day and 0.04 g/day, respectively. Compared to women of childbearing age included in our study, ALA intake in these women is twice as high while EPA and DHA intake would be fairly similar. In this regard, it is worth commenting the inherent difficulty of dietary records in estimating the intake of nutrients abundantly present in foods that are not consumed on a daily basis, such as seafood products, in terms of EPA and DHA intake. As previously mentioned, several studies acknowledge that median intake was much lower than the mean because of the intake of these LCPUFA is skewed and not normally distributed [43,44]. Nonetheless, it is worth noting that there is a trend of decreasing EPA and DHA intakes in younger age groups, both in the general population and in women of childbearing age, an observation that has also been confirmed in other studies [29,45]. This is consistent with the observation that the consumption of their major dietary sources, fish and shellfish, also follows the same trend, with mean daily intakes being significantly lower in children and adolescents compared to older age groups within the ANIBES population [46]. As a result, these foods provided approximately twice the total energy in the adult (3.7%) and elderly (4.7%) groups of the ANIBES study population compared to the child and adolescent groups (2.2% and 2.1%, respectively) [36].

Accordingly, the degree of non-compliance with nutritional goals set for omega-3 PUFA observed in the ANIBES Spanish population reflect a pattern of intakes again considerably less adequate than that reported by Ortega et al. [29], where the degree of inadequacy observed for the total of omega-3 was roughly a half to that reported here. Particularly striking is the different inadequacy levels reported for the combined intake of EPA + DHA (<0.25 g/day), which in the present study reached 83% of the population vs. only 35% in the study by Ortega et al. [29]. Furthermore, we did find a significantly higher degree of inadequacy in children and adolescents. For ALA, we observed insufficient intakes (<0.5% TE) in almost the entire sample of Spanish children (95%) and adolescents (90%). This degree of inadequacy contrasts with that reported in the HELENA study [47] carried out in 2008 in European adolescents, where 65% of them did reach sufficient ALA intakes. Nonetheless, data from the 2003–2014 National Health and Nutrition Examination Survey (NHANES) in the USA [48] also revealed that toddlers, children, and adolescents had significantly lower omega-3 PUFA intake (*p* < 0.001) when compared to adults and seniors. Evidence on the reduced intake of omega-3 PUFA in females, particularly at younger ages, highlights the importance of ensuring adequate supplies in pregnant and nursing women, given the potential implications of fetal reliance on maternal DHA. Unfortunately, pregnant women were one of the excluded population groups in the ANIBES study, so we could not assess intakes for this population group. However, median intakes observed for omega-3 PUFA in women of childbearing age among the ANIBES population were fairly similar to those observed in women of childbearing age included in the aforementioned NHANES survey (*n* = 8381; 20–44 years), which had mean intakes of 0.02 g/day for EPA and 0.05 g/day for DHA [48]. Interestingly, there were no significant differences in EPA, DHA, or combined EPA and DHA intake in pregnant women compared to non-pregnant women of childbearing age, despite the fact that the former showed significantly lower intakes of fish [48]. Supplementation strategies were proven relevant, as the difference in the supply of these fatty acids was provided by the use of EPA/DHA-containing supplements in 7.3% of pregnant women vs. 0.6% in non-pregnant women of childbearing-age. Nonetheless, mean intakes of EPA + DHA in pregnant women (*n* = 762) stood at 0.1 g/day, with DHA at 0.07 g/day [48], figures that are far from the respective 0.3 and 0.2 g/day proposed by the FAO/WHO guidelines [13]. Indeed, another NHANES analysis on data from years 2001–2014 found over 95% of U.S. women of childbearing age not meeting the combined EPA + DHA intake recommendation of 250 mg/day [49]. To conclude, a worldwide systematic review of data from 40 countries also confirms population groups of concern regarding insufficient EPA and DHA to be pregnant and nursing women along with infants, children, adolescents [50].

In the case of omega-6 fatty acids, the results obtained were very similar to those observed by Ortega et al. [29], except for AA, for which median intake was again reduced by half in the present study. At the European level, intakes were also similar for LA, however in the case of AA, median intakes were 0.2 g/day for French adults [43] but 0.04 g/day for Belgian women [44]. We observed higher intake levels of omega-6 PUFA in men, as reported for the French adult population, whereas in the study by Ortega et al., that observation was reported for women. In addition, observed intakes of AA remained constant across age groups in our study but increased with age in the study by Ortega et al. [29]. In contrast, both studies showed that average consumption of omega-6 and LA was higher among younger groups, which may also be explained by the higher meat consumption observed for them within the ANIBES Study [36], and which is probably being displaced by fish consumption at older ages.

Non-compliance with nutritional goals for omega-6 PUFA also showed discrepancies with previous evidence, although in this case the situation reported in the present study is slightly more positive. Hence, more than 95% of the population showed sufficient LA intakes (>2.5 g/day) vs. 87% in the study by Ortega et al. [29], where men also showed a significantly higher degree of inadequacy. We did not observe such differences according to gender, but we did find significant differences with respect to age, where the young population showed better compliance rates. The observations in this case are slightly better than those reported in the HELENA study [47], where 4.3% of European adolescents did not reach the minimum recommended intakes (vs. 2.4% in the present study). However, our results indicate that the focus should be placed on the elderly population, where the degree of inadequacy observed for LA intake was double than population average. A study on the US population from 2012–2015 [6,47] reported no such differences in LA intake by age, where children showed a mean intake comparable to this study of 13 g/day, but the elderly reached 15 g/day. These results suggest that special attention should be paid to the intake of this nutrient among the senior Spanish population as LA cannot be synthesized by the body and is therefore an essential fatty acid required to maintain metabolic integrity and cognitive function [13].

Consequently, the omega-6/omega-3 dietary ratio observed for the Spanish population demonstrates a situation of inadequacy that is even more marked than available evidence already showed [29]. Western-type diets tend to include insufficient omega-3 while omega-6 intake tends to be higher than recommended, as per the latest Reports on the Nutritional Assessment of the Spanish Diet according to the Food Consumption Panel, where the omega-6/omega-3 ratio of the Spanish population between 2000 and 2006 was approximately 16:1, dropping to 13:1 in 2008 [51]. The study by Ortega et al. [29], also from 2008, reported a ratio of 7:1. However, our results show a ratio that again rises in favor of omega-6 to 12:1, improving down to 9:1 in the elderly. The aforementioned study in the US population with intake data from 2012–2015 [6,47] reported slightly better ratios of 9:1 for children and 8:1 for the elderly, demonstrating that a Western-type diet would not necessarily cause this ratio to spike. Indeed, the HELENA study on European adolescents [47] described a 6:1 ratio that did not differ significantly between boys and girls. It should be noted that other confounding factors may be affecting the observed intakes, as in the present study individuals presenting obesity showed a significantly lower intake of omega-6 and thus a significantly lower ratio than underweight individuals, but not than those with normal or overweight BMI. Another observational study in 209 Spanish children and adolescents reported no significant differences in this intake ratio according to BMI [7]. Another factor we took into account was the geographical residence of the population, for which we observed no significant differences. In addition, we acknowledge that socioeconomic factors undoubtedly influence food choices, but we have not proceeded to include this analysis because of the heterogenous response rate obtained for questions related to household income.

Neither the Spanish Society of Community Nutrition (SENC) nor the EFSA [1] or the FAO/WHO expert panel [13] have established any recommendation regarding the omega-6/omega-3 ratio, deeming it redundant if intakes of both PUFA series lie within the recommendations. However, available evidence support ratios below 10 to be more optimal [6] as higher ratios could lead to greater concentrations of AA in cell membranes, thus hindering the synthesis of anti-inflammatory mediators from EPA and DHA. Although several studies have associated high omega-6/omega-3 ratios with a greater risk of cancer [52,53], this association has not been observed for high intakes of omega-6 alone, therefore current recommendations focus on the absolute intake of EPA and DHA [13]. The results obtained in the present study also suggest that the strategy to improve the dietary ratio should focus on improving intakes of omega-3 PUFA, for which we found high levels of inadequacy in the whole population. Meanwhile, adequacy observed for omega-6 intakes is relatively acceptable and would not justify an intervention in this respect. Indeed, different studies suggest that moderate intakes of omega-6 PUFA LA (5–8% TE) do not lead to higher elevations of AA and therefore do not increase the formation of pro-inflammatory mediators associated to the intake of omega-6 PUFA [13,54].

Another index of diet quality is the EPA/DHA ratio, which in the present study was higher (0.57 vs. 0.33) than that reported by Ortega et al. [29]. This parameter is also relevant as some studies have pointed out that there is a great variability in the EPA/DHA ratio in diets and supplements, and that its modification can have a specific influence on various cardiovascular or neurological pathologies [55,56]. However, no specific recommendations have been issued for the time being. Considering the FAO/WHO recommendation for adult pregnant and lactating females, 0.3 g/day EPA + DHA with at least 0.2 g/day of DHA [13], a recommended EPA/DHA ratio of 1.5 could be derived. It is apparent that the EPA/DHA ratio found in the present study is probably somewhat lower than that figure and also than those found in studies that have found various health benefits [55,56].

### 4.2. Omega-3 and Omega-6 PUFA Dietary Sources

Remarkably, both fish and shellfish and meat and meat products contributed in a similar degree to total omega-3 intakes among the ANIBES Spanish population. Although closely followed by meat and meat products, in the work by Ortega et al. [29], fish and shellfish remained the main source of omega-3 PUFA in the Spanish population, a pattern that was now only observed for the elderly population of the ANIBES Study. In contrast, for adults, the contribution of both food groups to total omega-3 intakes was virtually the same. Moreover, in children and adolescents meat and meat products have displaced fish and shellfish as the main source of their omega-3 PUFA intakes, contributing significantly more than in older age groups, while fish and shellfish contributed significantly less. As it is—or perhaps it was—characteristic of Mediterranean populations, the Spanish population has usually showed a high seafood consumption, higher than other Western countries [57] and only lower than that of Japan [58]. However, fish and shellfish consumption has decreased nearly 30% in the past few years in Spain, mostly for the youngest population groups. This decline in fish and shellfish consumption could explain the worsening of the situation regarding insufficient omega-3 intakes compared to previous findings [29]. Indeed, within the ANIBES population, daily mean consumption of meat and mead products was more than twice that of fish and shellfish, with children and adolescents showing significantly higher intakes of meat and meat products, and significantly less for fish and shellfish, compared to older groups [46]. Likewise, in the HELENA study [47], meat and meat products contributed almost twice as much as fish and shellfish to the total omega-3 intake of European adolescents. However, it is imperative to highlight that in our study meat and meat products only provided omega-3 PUFA in the form of ALA, which wouldn’t ensure an adequate nutritional status of EPA and/or DHA because of the very limited metabolic conversion from dietary ALA [8]. This is precisely why the analysis of intakes and dietary sources of individual PUFAs, rather than omega-3 series as a whole, is of special relevance.

Regarding dietary intakes of EPA and DHA, fish and shellfish were the major and almost exclusive contributors to the observed intakes in all age groups. Likewise, in the European population from the HELENA study [47] fish and shellfish were the main source of EPA and DHA; and other studies in the European adult population also found similar results [43,44]. As discussed above with regard to conversion from ALA, strategies to increase EPA and DHA intake must necessarily involve an increase in fish and shellfish consumption, or alternatively, supplements as a necessary substitute.

Analyzing the observed dietary sources of ALA, it is perhaps this fatty acid for which we have found the greatest differences with other populations. Meat and meat products together with oils and fats are observed to be the largest contributors for the Spanish population, something that varies from other European populations, where meat and meat products were not as relevant. In French adults, dairy was the main food source and oils and fats contribution was minor, while in Belgian women, fats and oils provided almost half of the ALA consumed [43,44].

With regard to omega-6 PUFA, the main dietary sources and their contributions are very similar to those described by Ortega et al. [29] in the Spanish population, and also to the available European studies [43,44]. It is worth mentioning that in adolescents in the HELENA [47] study the contribution of oils and fats to total omega-6 and LA intakes was much lower. In contrast, the consumption of oils and fats observed in the Spanish population could represent an interesting strategy to increase the EPA and DHA status, as some reports have suggested that EPA and DHA synthesis could be enhanced by long-term intakes of vegetable oils containing more ALA and less LA (rapeseed, soybean, or walnut oils) [59,60]. This observation could be of great interest for population groups that consume less fish, such as young people and vegetarian or vegan groups, given that in the Spanish population the contribution of oils and fats to ALA intake is already important. However, as discussed above, the conversion of ALA to EPA and DHA is limited and varies greatly among populations [8]. Moreover, this strategy would not be optimal for the Spanish senior population, for which significantly lower intakes of LA were observed. On the other hand, we would like to emphasize the fact that lipid intake must also be globally balanced, and not only with regard to the omega-3 and 6 series of PUFA. Therefore, overall attention should be paid to dietary patterns when suggesting strategies such as modifying vegetable oils intake, considering that one positive aspect of the observed patterns among the Spanish population from the ANIBES Study is the relatively high contribution of monounsaturated fatty acids (MUFA), which is mostly owed to the common use of olive oil [36].

### 4.3. Combined Dietary Intake Adequacy of Omega-3 PUFA, Folic Acid (FA), Vitamin B_12_ and Choline

The present study confirms the need to raise awareness about optimizing the status of these nutrients, where more than half of the Spanish population would not consume enough omega-3 PUFA and FA; and a quarter neither omega-3 PUFA and vitamin B₁₂ nor omega-3 PUFA and choline. We have already mentioned possible functions in which all these nutrients could jointly influence, although by different mechanisms (prevention of cerebrovascular events, development, and maintenance of cognitive function) and therefore the possible consequences of an inadequate combined intake of omega-3 PUFA with FA, vitamin B₁₂ and choline cannot be ignored when aiming to improve the nutritional status of the population. Probably some of the positive effects associated with sufficient intakes of omega-3 fatty acids (anti-hyperlipidemia, anti-inflammatory, anti-hypertensive, or anti-thrombotic effects [61]) cannot be fully exerted without ensuring the correct nutritional status of other micronutrients such as those proposed in the present work. Therefore, all these data support the need to consider the convenience of combined supplements/multivitamins, to help guarantee the adequate intake of all these nutrients along different life stages, including pregnancy and lactation.

### 4.4. Strengths and Limitations

Participant and social desirability biases are inherent to the use of dietary records, which however are considered a reference method for validation studies [62]. In addition, as already mentioned, it is necessary to acknowledge the difficulty of estimating the dietary intake of nutrients that are abundantly present in foods which are usually not consumed on a daily basis, such as seafood products for EPA and DHA.

Despite such limitations, the ANIBES study aimed to provide the most reliable results on evaluate dietary habits and its determinants for the Spanish population by targeting a representative national sample from a broad age range (9–75 years) and by using innovative and novel tools to accurately record both actual dietary intakes and leftovers.

## 5. Conclusions

In light of the high percentage of Spanish individuals who do not meet the nutritional goals set for omega-3 PUFA, ALA, EPA, and DHA, it is advisable to increase the consumption of fish and/or foods enriched and/or nutritional supplements with these fatty acids. This would be of special relevance during pregnancy and lactation, but also the elderhood, given the roles of EPA and DHA in neurological and visual development and function. These efforts should also represent the main strategy for the optimization of the omega-6/omega-3 ratio, as the adequacy observed for omega-6 PUFA intakes among the Spanish population is relatively acceptable.

Results highlight the urgent need to prioritize and discuss nutritional supplementation policies for all these components, following the approach taken with folic acid, and considering both benefits and risks. This would facilitate compliance with individual requirements in a prompt and effective manner while stressing the importance of improving dietary intakes of key nutrients such as those considered here.

## Figures and Tables

**Table 1:** Omega-3 fatty acid dietary intake among the ANIBES Study population.

			*n*	Omega-3 Fatty Acids (ω3) (g/Day)	Alfa-Linolenic Acid (ALA) (C18: 3*n*-3) (g/Day)	Eicosapentaenoic Acid (EPA) (C20: 5*n*-3) (g/Day)	Docosahexaenoic Acid (DHA) (C22: 6*n*-3) (g/Day)	EPA + DHA (g/Day)
Total Population (9–75 years)		Total	2009	0.81	0.61	0.03	0.06	0.09
				(0.56–1.19)	(0.45–0.85)	(0.01–0.12)	(0.01–0.20)	(0.02–0.34)
		Men	1013	0.86 ***	0.65 ***	0.03	0.06	0.10
				(0.60–1.26)	(0.50–0.89)	(0.01–0.12)	(0.01–0.22)	(0.02–0.36)
		Women	996	0.75	0.57	0.03	0.06	0.09
				(0.52–1.11)	(0.42–0.79)	(0.01–0.11)	(0.01–0.19)	(0.03–0.33)
Age	9–12 years	Total	213	0.77	0.61	0.03 ^a,b^	0.05 ^a,b^	0.09 ^a,b,c^
				(0.57–1.02)	(0.48–0.79)	(0.01–0.10)	(0.01–0.17)	(0.02–0.26)
		Men	126	0.81	0.62	0.03	0.05	0.10
				(0.61–1.02)	(0.49–0.82)	(0.01–0.09)	(0.01–0.19)	(0.02–0.29)
		Women	87	0.72	0.57	0.04	0.05	0.09
				(0.55–1.02)	(0.46–0.78)	(0.01–0.10)	(0.01–0.13)	(0.02–0.22)
	13–17 years	Total	211	0.80	0.63	0.02 ^b^	0.04 ^a^	0.06 ^b^
				(0.55–1.10)	(0.50–0.90)	(0.01–0.07)	(0.01–0.12)	(0.01–0.21)
		Men	137	0.85 **	0.67 ***	0.02	0.04	0.07
				(0.59–1.14)	(0.54–0.98)	(0.01–0.08)	(0.01–0.13)	(0.01–0.21)
		Women	74	0.68	0.54	0.02	0.03	0.05
				(0.49–1.04)	(0.45–0.75)	(0.01–0.07)	(0.00–0.09)	(0.02–0.19)
	18–64 years	Total	1655	0.82	0.62	0.03 ^b^	0.06 ^b^	0.09 ^a,c^
				(0.56–1.20)	(0.46–0.86)	(0.01–0.12)	(0.01–0.20)	(0.02–0.34)
		Men	798	0.87 ***	0.66 ***	0.03	0.06	0.09
				(0.60–1.27)	(0.50–0.90)	(0.01–0.12)	(0.01–0.21)	(0.02–0.36)
		Women	857	0.76	0.58	0.03	0.05	0.09
				(0.53–1.12)	(0.42–0.79)	(0.01–0.11)	(0.01–0.20)	(0.02–0.33)
	65–75 years	Total	206	0.81	0.58	0.05 ^a^	0.09 ^c^	0.15 ^d^
				(0.55–1.25)	(0.41–0.82)	(0.01–0.15)	(0.03–0.25)	(0.05–0.39)
		Men	99	0.95 **	0.61 *	0.06 **	0.13 *	0.18
				(0.62–1.55)	(0.45–0.84)	(0.01–0.19)	(0.03–0.30)	(0.05–0.48)
		Women	107	0.79	0.50	0.04	0.08	0.13
				(0.51–1.08)	(0.38–0.79)	(0.01–0.09)	(0.03–0.17)	(0.04–0.26)

Values are median (interquartile range) per group. Different superscript lowercase letters (^a^, ^b^, ^c^ and ^d^) indicate statistically significant difference in each row between age groups (all differences are *p* ≤ 0.05; Kruskal–Wallis and Dunn tests to adjust for multiple comparison and Bonferroni’s correction to adjust the *p*-value) and asterisks indicate statistically significant difference between gender (* *p* ≤ 0.05, ** *p* ≤ 0.01, *** *p* ≤ 0.001; Mann–Whitney U test).

**Table 2:** Omega-3 fatty acid dietary intake among women of two childbearing age groups from the ANIBES Study.

	*n*	Omega-3 Fatty Acids (ω3) (g/Day)	Alfa-Linolenic Acid (ALA) (C18: 3*n*-3) (g/Day)	Eicosapentaenoic Acid (EPA) (C20: 5*n*-3) (g/Day)	Docosahexaenoic Acid (DHA) (C22: 6*n*-3) (g/Day)	EPA + DHA (g/Day)
Total women of childbearing age	552	0.74	0.58	0.02	0.05	0.07
		(0.51–1.04)	(0.42–0.77)	(0.01–0.10)	(0.01–0.18)	(0.02–0.28)
Younger women (18–30 years)	209	0.69 *	0.56	0.01 ***	0.03 **	0.05 **
		(0.49–0.95)	(0.42–0.77)	(0.00–0.07)	(0.01–0.14)	(0.02–0.22)
Older women (31–45 years)	343	0.76	0.59	0.03	0.06	0.09
		(0.53–1.09)	(0.41–0.79)	(0.01–0.12)	(0.01–0.20)	(0.03–0.33)

Values are median (interquartile range) per group. Asterisks indicate statistically significant difference between age groups (* *p* ≤ 0.05, ** *p* ≤ 0.01, *** *p* ≤ 0.001; Mann–Whitney U test).

**Table 3:** Omega-6 fatty acid dietary intake among the ANIBES Study population.

			*n*	Omega-6 Fatty Acids (ω6) (g/Day)	Linoleic Acid (la) (c18: 2*n*-6) (g/Day)	Arachidonic Acid (aa) (c20: 4*n*-6) (g/Day)
Total Population (9–75 years)		Total	2009	10.07	10.00	0.08
				(7.00–14.01)	(6.93–13.90)	(0.05–0.13)
		Men	1013	10.78 ***	10.69 ***	0.09 ***
				(7.65–14.86)	(7.53–14.75)	(0.06–0.14)
		Women	996	9.16	9.07	0.07
				(6.41–12.78)	(6.34–12.73)	(0.04–0.11)
Age	9–12 years	Total	213	11.58 ^a^	11.51 ^a^	0.09 ^a^
				(8.63–14.52)	(8.52–14.42)	(0.06–0.13)
		Men	126	11.64	11.53	0.09
				(8.92–14.49)	(8.82–14.36)	(0.06–0.14)
		Women	87	11.44	11.27	0.09
				(8.12–14.84)	(8.02–14.76)	(0.06–0.13)
	13–17 years	Total	211	11.33 ^b^	11.16 ^a^	0.09 ^a^
				(8.07–16.13)	(7.95–16.00)	(0.06–0.13)
		Men	137	12.75 *	12.60 *	0.10 **
				(9.21–16.21)	(9.06–16.04)	(0.07–0.14)
		Women	74	10.23	10.21	0.07
				(7.32–15.77)	(7.21–15.70)	(0.05–0.12)
	18–64 years	Total	1655	10.18 ^c^	10.10 ^b^	0.08 ^b^
				(7.16–14.12)	(7.06–14.04)	(0.05–0.13)
		Men	798	10.88	10.75 ***	0.09 ***
				(7.72–15.02) ***	(7.67–14.88)	(0.06–0.14)
		Women	857	9.35	9.25	0.07
				(6.62–12.94)	(6.53–12.83)	(0.04–0.11)
	65–75 years	Total	206	7.91 ^d^	7.79 ^c^	0.07 ^b^
				(5.22–11.25)	(5.13–11.19)	(0.04–0.11)
		Men	99	8.24	8.15	0.09 ***
				(5.87–11.98)	(5.68–11.92)	(0.06–0.14)
		Women	107	7.73	7.66	0.06
				(4.69–11.13)	(4.60–10.95)	(0.04–0.09)

Values are median (interquartile range) per group. Different superscript lowercase letters (^a^, ^b^, ^c^ and ^d^) indicate statistically significant difference in each row between age groups (all differences are *p* ≤ 0.05; Kruskal–Wallis and Dunn tests to adjust for multiple comparison and Bonferroni’s correction to adjust the *p*-value) and asterisks indicate statistically significant difference between gender (* *p* ≤ 0.05, ** *p* ≤ 0.01, *** *p* ≤ 0.001; Mann–Whitney U test).

**Table 4:** Omega-6 fatty acid dietary intake among women of two childbearing age groups from the ANIBES Study.

	*n*	Omega-6 Fatty Acids (ω6) (g/Day)	Linoleic Acid (LA) (C18: 2*n*-6) (g/Day)	Arachidonic Acid (AA) (C20: 4*n*-6) (g/Day)
Total women of childbearing age	552	9.88	9.74	0.07
		(6.85–13.65)	(6.80–13.53)	(0.04–0.11)
Younger women (18–30 years)	209	9.93	9.76	0.07
		(7.26–13.42)	(7.20–13.42)	(0.04–0.11)
Older women (31–45 years)	343	9.84	9.70	0.08
		(6.73–13.79)	(6.70–13.68)	(0.05–0.12)

Values are presented as median (interquartile range) per group.

**Table 5:** Omega-6/Omega-3 and EPA/DHA dietary intake ratios among the ANIBES Study population.

			*n*	Omega-6/Omega-3	EPA/DHA
Total Population (9–75 years)		Total	2009	12.13	0.57
				(8.69–16.32)	(0.32–0.94)
		Men	1013	12.40 *	0.58
				(8.89–16.67)	(0.32–0.95)
		Women	996	11.83	0.57
				(8.57–15.89)	(0.31–0.93)
Age	9–12 years	Total	213	14.30 ^a^	0.61
				(10.94–18.86)	(0.35–1.08)
		Men	126	13.89	0.61
				(11.26–18.84)	(0.31–1.10)
		Women	87	14.99	0.61
				(10.76–18.90)	(0.38–1.06)
	13–17 years	Total	211	14.24 ^a^	0.62
				(10.76–19.11)	(0.41–1.13)
		Men	137	14.06	0.62
				(10.64–18.52)	(0.44–1.06)
		Women	74	14.74	0.52
				(10.89–21.50)	(0.32–1.49)
	18–64 years	Total	1655	12.22 ^b^	0.56
				(8.87–16.37)	(0.30–0.96)
		Men	798	12.59 *	0.56
				(8.92–16.90)	(0.28–0.96)
		Women	857	11.94	0.56
				(8.79–15.83)	(0.30–0.95)
	65–75 years	Total	206	9.36 ^c^	0.59
				(6.50–12.63)	(0.34–0.88)
		Men	99	9.18	0.60
				(5.94–12.22)	(0.38–0.84)
		Women	107	9.58	0.54
				(7.31–13.12)	(0.23–0.89)

EPA: eicosapentaenoic acid; DHA: docosahexaenoic acid. Values are median (interquartile range) per group. Different superscript lowercase letters (^a^, ^b^ and ^c^) indicate statistically significant difference in each row between age groups (all differences are *p* ≤ 0.05; Kruskal–Wallis and Dunn tests to adjust for multiple comparison and Bonferroni’s correction to adjust the *p*-value) and asterisks indicate statistically significant difference between gender (* *p* ≤ 0.05; Mann–Whitney U test).

**Table 6:** Omega-6/Omega-3 and EPA/DHA dietary intake ratios among women of two childbearing age groups from the ANIBES Study.

	*n*	Omega-6/Omega-3	EPA/DHA
Total women of childbearing age	552	12.78	0.56
		(9.61–17.52)	(0.29–1.04)
Younger women (18–30 years)	209	13.86 ***	0.50
		(10.60–18.13)	(0.23–0.97)
Older women (31–45 years)	343	11.94	0.59
		(8.67–16.08)	(0.34–1.08)

EPA: eicosapentaenoic acid; DHA: docosahexaenoic acid. Values are median (interquartile range) per group. *** *p* ≤ 0.001 respect to older women; Mann–Whitney U test.

**Table 7:** Omega-3 and omega-6 fatty acid dietary intake and ratios according to body weight among the ANIBES Study population.

		Total Population (9–75 Years)			
		Omega-3 Fatty Acids (ω3) (g/Day)	Omega-6 Fatty Acids (ω6) (g/Day)	Omega-6/Omega-3	EPA/DHA
BMI	Underweight *n* = 42	0.74	10.40 ^a^	13.88 ^a^	0.62
		(0.56–0.93)	(8.05–14.97)	(11.70–17.26)	(0.47–1.46)
	Normal *n* = 854	0.84	10.68 ^a,b^	12.20 ^a,b^	0.58
		(0.58–1.19)	(7.39–14.58)	(8.94–16.66)	(0.32–0.94)
	Overweight *n* = 713	0.80	9.86 ^a,b^	12.12 ^a,b^	0.59
		(0.56–1.21	(6.85–13.88)	(8.56–16.09)	(0.32–1.00)
	Obesity *n* = 400	0.77	9.15 ^b^	11.85 ^b^	0.51
		(0.53–1.21)	(6.67–12.86)	(8.42–15.83)	(0.29–0.83)

EPA: eicosapentaenoic acid; DHA: docosahexaenoic acid. Values are median (interquartile range) per group. Different superscript lowercase letters (^a^, and ^b^) indicate statistically significant difference in each row between groups (all differences are *p ≤* 0.05; Kruskal–Wallis and Dunn tests to adjust for multiple comparison and Bonferroni’s correction to adjust the *p*-value).

**Table 8:** Omega-3 and omega-6 fatty acid dietary intake and ratios according to geographical areas (Nielsen regions) of residence among the ANIBES Study population.

	Total Population (9–75 Years)			
	Omega-3 Fatty Acids (ω3) (g/Day)	Omega-6 Fatty Acids (ω6) (g/Day)	Omega-6/Omega-3	EPA/DHA
Barcelona (Metropolitan Area) *n* = 129	0.82	9.64	10.96	0.59
	(0.56–1.45)	(7.47–12.96)	(7.87–15.79)	(0.42–0.89)
Canary Islands *n* = 93	0.71	8.77	11.80	0.46
	(0.53–1.15)	(6.77–11.60)	(8.36–15.85)	(0.23–1.55)
Central *n* = 191	0.81	10.05	12.44	0.59
	(0.59–1.16)	(6.73–13.99)	(8.76–17.55)	(0.37–0.84)
Levante (East) *n* = 335	0.87	10.71	11.78	0.55
	(0.55–1.33)	(6.87–14.79)	(8.52–15.88)	(0.32–0.74)
Madrid (Metropolitan Area) *n* = 264	0.77	9.51	11.86	0.62
	(0.54–1.13)	(6.59–13.30)	(8.59–15.74)	(0.38–0.88)
Northeast *n* = 240	0.74	10.57	12.98	0.59
	(0.56–1.10)	(7.65–13.93)	(9.95–17.51)	(0.33–0.87)
Northwest *n* = 152	0.81	9.66	11.62	0.58
	(0.55–1.32)	(6.54–14.29)	(8.16–15.88)	(0.27–1.44)
North Central *n* = 162	0.90	11.00	12.08	0.59
	(0.60–1.24)	(7.29–15.18)	(8.67–15.88)	(0.31–1.27)
South *n* = 443	0.82	9.93	12.73	0.50
	(0.56–1.14)	(7.14–14.22)	(8.94–16.54)	(0.24–1.01)

EPA: eicosapentaenoic acid; DHA: docosahexaenoic acid. Values are median (interquartile range) per group.

**Table 9:** Degree of non-compliance with nutritional goals for polyunsaturated fatty acids (PUFA) among the ANIBES Study population.

	Nutritional Goals ^1^	Non-Compliance with Nutritional Goals	Total	Men	Women	9–12 Years	13–17 Years	18–64 Years	65–75 Years
			*n* = 2009	*n* = 1013	*n* = 996	*n* = 213	*n* = 211	*n* = 1655	*n* = 206
Omega-3 PUFA:									
Total Omega-3 PUFA (%)	0.5–2% TE	<0.5% TE	65.1	65.6	64.5	78.9 ^a^	78.2 ^a^	64.5 ^b^	54.9 ^c^
		>2% TE	0.4	0.5	0.4	0.0	0.0	0.5	0.5
Alfa-Linolenic acid (ALA) (%)	>0.5% TE	<0.5% TE	87.0	87.8	86.2	95.3 ^a^	90.0 ^a^	87.0 ^b^	81.6 ^c^
EPA + DHA (%)	0.25–2 g/day	<0.25 g/day	83.3	82.3	84.3	88.3 ^a,c^	90.5 ^b^	83.1 ^c^	83.0 ^c^
		>2 g/day	1.0	1.3	0.8	0.0	0.5	1.1	1.0
Omega-6 PUFA:									
Linoleic acid (LA) (%)	2.5–9% TE	<2.5% TE	4.5	4.0	5.0	2.3 ^a^	2.4 ^a^	4.0 ^a^	10.2 ^b^
		>9% TE	5.9	5.8	5.9	5.2	6.2	6.0	5.3

^1^ FAO/WHO dietary recommendations [13]. Different superscript lowercase letters (^a^, ^b^ and ^c^) indicate statistically significant difference in each row between age groups (all differences are *p* ≤ 0.05; Kruskal–Wallis and Dunn tests to adjust for multiple comparison and Bonferroni’s correction to adjust the *p*-value).

**Table 10:** Degree of non-compliance with nutritional goals for polyunsaturated fatty acids (PUFA) among women of two childbearing age groups from the ANIBES Study.

	Nutritional Goals ^1^	Non-Compliance with Nutritional Goals	Total Women of Childbearing Age (18–45 Years)	Younger Women (18–30 Years)	Older Women (31–45 Years)
			*n* = 552	*n* = 209	*n* = 343
Omega-3 PUFA:					
Total Omega-3 PUFA (%)	0.5–2% TE	<0.5% TE	69.6	75.1 *	66.2
		>2% TE	0.2	0.0	0.3
Alfa-Linolenic acid (ALA) (%)	>0.5% TE	<0.5% TE	88.9	90.9	88.7
EPA + DHA (%)	0.25–2 g/day	<0.25 g/day	83.5	90.0	84.5
		>2 g/day	1.0	0.5	0.9
Omega-6 PUFA:					
Linoleic acid (LA) (%)	2.5–9% TE	<2.5% TE	4.0	3.8	4.1
		>9% TE	6.5	7.7	5.8

^1^ FAO/WHO dietary recommendations [13]. * *p* ≤ 0.05 respect to older women; Mann–Whitney U test.

**Table 11:** Contribution of dietary food sources to lipid intakes among the ANIBES Study population.

Food Sources (g/Day)	Lipids	SFA	MUFA	PUFA
Oils and fats	22.7	3.9	13.3	3.7
	(16.7–29.6)	(2.8–5.6)	(9.6–17.6)	(3.2–6.2)
Meat and meat products	16.5	5.8	7.1	2.1
	(9.8–25.4)	(3.3–9.0)	(4.1–10.9)	(1.3–3.3)
Cereals and derivatives	6.3	1.9	1.8	1.2
	(3.0–11.2)	(0.7–3.8)	(0.8–3.3)	(0.7–2.3)
Fish and shellfish	1.9	0.3	0.4	0.5
	(0.1–4.7)	(0.0–0.9)	(0.0–1.4)	(0.0–2.2)
Eggs	2.2	0.6	0.8	0.3
	(0.4–4.2)	(0.1–1.2)	(0.1–1.5)	(0.1–0.6)
Milk and dairy products	9.1	5.2	2.3	0.2
	(5.5–13.5)	(3.2–7.9)	(1.4–3.5)	(0.1–0.4)
Vegetables	0.3	0.0	0.0	0.1
	(0.1–0.5)	(0.0–0.1)	(0.0–0.0)	(0.1–0.2)
Ready-to-eat meals	0.7	0.1	0.2	0.1
	(0.0–5.7)	(0.0–1.9)	(0.0–2.2)	(0.0–0.9)
Sauces and condiments	0.8	0.1	0.2	0.0
	(0.0–3.7)	(0.0–0.5)	(0.0–2.1)	(0.3–0.8)
Fruits	0.1	0.0	0.0	0.0
	(0.0–0.7)	(0.0–0.1)	(0.0–0.2)	(0.0–0.2)
Legumes	0.0	0.0	0.0	0.0
	(0.0–0.4)	(0.0–0.0)	(0.0–0.0)	(0.0–0.2)
Appetizers	0.0	0.0	0.0	0.0
	(0.0–0.9)	(0.0–0.1)	(0.0–0.6)	(0.0–0.1)
Sugars and sweets	0.2	0.1	0.1	0.0
	(0.0–1.4)	(0.0–0.7)	(0.0–0.4)	(0.0–0.1)
Supplements and meal replacement	0.0	0.0	0.0	0.0
	(0.0–0.0)	(0.0–0.0)	(0.0–0.0)	(0.0–0.0)
Non-alcoholic beverages	0.0	0.0	0.0	0.0
	(0.0–0.1)	(0.0–0.0)	(0.0–0.0)	(0.0–0.0)
Alcoholic beverages	0.0	0.0	0.0	0.0
	(0.0–0.0)	(0.0–0.0)	(0.0–0.0)	(0.0–0.0)

SFA: saturated fatty acids; MUFA: monounsaturated fatty acids; PUFA: polyunsaturated fatty acids. Values are median (interquartile range) per group.

**Table 12:** Contribution of dietary food sources to omega-3 fatty acid intakes among the ANIBES Study population.

Food Sources (g/Day)	Omega-3 Fatty Acids (ω3)	Alfa-linolenic Acid (ALA) (C18: 3*n*-3)	Eicosapentaenoic Acid (EPA) (C20: 5*n*-3)	Docosahexaenoic Acid (DHA) (C22: 6*n*-3)
Oils and fats	0.09	0.09	0.00	0.00
	(0.06–0.14)	(0.06–0.14)	(0.00–0.00)	(0.00–0.00)
Meat and meat products	0.15	0.14	0.00	0.00
	(0.08–0.23)	(0.08–0.23)	(0.00–0.00)	(0.00–0.00)
Cereals and derivatives	0.07	0.03	0.00	0.00
	(0.07–0.17)	(0.07–0.17)	(0.00–0.00)	(0.00–0.00)
Fish and shellfish	0.15	0.03	0.03	0.05
	(0.01–0.41)	(0.00–0.12)	(0.00–0.10)	(0.00–0.20)
Eggs	0.03	0.03	0.00	0.00
	(0.00–0.06)	(0.00–0.06)	(0.00–0.00)	(0.00–0.00)
Milk and dairy products	0.04	0.04	0.00	0.00
	(0.02–0.07)	(0.02–0.06)	(0.00–0.00)	(0.00–0.00)
Vegetables	0.01	0.01	0.00	0.00
	(0.00–0.03)	(0.00–0.03)	(0.00–0.00)	(0.00–0.00)
Ready-to-eat meals	0.00	0.00	0.00	0.00
	(0.00–0.03)	(0.00–0.02)	(0.00–0.00)	(0.00–0.00)
Sauces and condiments	0.00	0.00	0.00	0.00
	(0.00–0.02)	(0.00–0.02)	(0.00–0.00)	(0.00–0.00)
Fruits	0.00	0.00	0.00	0.00
	(0.00–0.02)	(0.00–0.02)	(0.00–0.00)	(0.00–0.00)
Legumes	0.00	0.00	0.00	0.00
	(0.00–0.00)	(0.00–0.00)	(0.00–0.00)	(0.00–0.00)
Appetizers	0.00	0.00	0.00	0.00
	(0.00–0.00)	(0.00–0.00)	(0.00–0.00)	(0.00–0.00)
Sugars and sweets	0.00	0.00	0.00	0.00
	(0.00–0.00)	(0.00–0.00)	(0.00–0.00)	(0.00–0.00)
Supplements and meal replacement	0.00	0.00	0.00	0.00
	(0.00–0.00)	(0.00–0.00)	(0.00–0.00)	(0.00–0.00)
Non-alcoholic beverages	0.00	0.00	0.00	0.00
	(0.00–0.00)	(0.00–0.00)	(0.00–0.00)	(0.00–0.00)
Alcoholic beverages	0.00	0.00	0.00	0.00
	(0.00–0.00)	(0.00–0.00)	(0.00–0.00)	(0.00–0.00)

Values are median (interquartile range) per group.

**Table 13:** Contribution of dietary food sources to total omega-3 intakes per age group among the ANIBES Study population.

Food Sources (g/Day)	Children	Adolescents	Adults	Elderly
Meat and meat products	0.16 ^a^	0.18 ^a^	0.15 ^b^	0.12 ^c^
	(0.11–0.24)	(0.12–0.26)	(0.08–0.23)	(0.06–0.19)
Fish and shellfish	0.11 ^a^	0.11 ^a^	0.15 ^b^	0.17 ^b^
	(0.00–0.31)	(0.00–0.33)	(0.01–0.41)	(0.03–0.42)
Oils and fats	0.09 ^a^	0.08 ^a^	0.09 ^a^	0.11 ^b^
	(0.06–0.13)	(0.05–0.12)	(0.06–0.14)	(0.07–0.16)
Cereals and derivatives	0.07 ^a^	0.09 ^a^	0.06 ^b^	0.05 ^b^
	(0.07–0.16)	(0.04–0.20)	(0.03–0.18)	(0.02–0.14)
Milk and dairy products	0.06 ^a^	0.05 ^a^	0.04 ^b^	0.03 ^c^
	(0.03–0.08)	(0.02–0.07)	(0.01–0.06)	(0.01–0.05)
Eggs	0.03	0.03	0.03	0.03
	(0.00–0.06)	(0.00–0.05)	(0.00–0.05)	(0.01–0.06)
Vegetables	0.01 ^a^	0.01 ^a^	0.01 ^a^	0.02 ^b^
	(0.00–0.02)	(0.00–0.02)	(0.00–0.03)	(0.00–0.04)

Values are median (interquartile range) per group. Different superscript lowercase letters (^a^, ^b^ and ^c^) indicate statistically significant difference between age groups (all differences are *p* ≤ 0.05; Kruskal–Wallis and Dunn tests to adjust for multiple comparison and Bonferroni’s correction to adjust the *p*-value).

**Table 14:** Contribution of dietary food sources to α-linolenic acid (ALA) intakes per age group among the ANIBES Study population.

Food Sources (g/Day)	Children	Adolescents	Adults	Elderly
Meat and meat products	0.15 ^a,b^	0.18 ^b^	0.14 ^a^	0.11 ^c^
	(0.11–0.23)	(0.12–0.25)	(0.08–0.23)	(0.06–0.19)
Oils and fats	0.09 ^a^	0.08 ^a^	0.09 ^a^	0.11 ^b^
	(0.06–0.13)	(0.05–0.13)	(0.06–0.14)	(0.07–0.15)
Cereals and derivatives	0.08 ^a,b^	0.10 ^b^	0.07 ^a^	0.05 ^c^
	(0.05–0.16)	(0.04– 0.20)	(0.03–0.18)	(0.02–0.13)
Milk and dairy products	0.05 ^a^	0.05 ^a^	0.04 ^b^	0.03 ^c^
	(0.03–0.08)	(0.02–0.07)	(0.02–0.06)	(0.01–0.05)
Fish and shellfish	0.01	0.01	0.03	0.03
	(0.00–0.09)	(0.00–0.10)	(0.00–0.12)	(0.00–0.10)
Eggs	0.03	0.03	0.03	0.03
	(0.00–0.06)	(0.00–0.05)	(0.00–0.06)	(0.01–0.06)
Vegetables	0.01 ^a^	0.01 ^a^	0.01 ^b^	0.02 ^b^
	(0.00–0.02)	(0.00–0.03)	(0.00–0.03)	(0.00–0.04)
Ready-to-eat meals	0.00 ^a,b^	0.01 ^a^	0.00 ^b^	0.00 ^b^
	(0.00–0.03)	(0.00–0.04)	(0.00–0.02)	(0.00–0.02)

Values are median (interquartile range) per group. Different superscript lowercase letters (^a^, ^b^ and ^c^) indicate statistically significant difference between age groups (all differences are *p* ≤ 0.05; Kruskal–Wallis Test and the Dunn to adjust for multiple comparison and adjust the *p*-value with Bonferroni’s correction).

**Table 15:** Contribution of dietary food sources to eicosapentaenoic acid (EPA) intakes per age group among the ANIBES Study population.

Food Sources (g/Day)	Children	Adolescents	Adults	Elderly
Fish and shellfish	0.011 ^a^	0.002 ^a^	0.023 ^b^	0.033 ^b^
	(0.000–0.070)	(0.000–0.080)	(0.000–0.106)	(0.000–0.112)
Milk and dairy products	0.002 ^a^	0.002 ^a,b^	0.001 ^b^	0.001 ^b^
	(0.001–0.003)	(0.001–0.003)	(0.001–0.002)	(0.000–0.002)

Values are median (interquartile range) per group. Different superscript lowercase letters (^a^ and ^b^) indicate statistically significant difference between age groups (all differences are *p* ≤ 0.05; Kruskal–Wallis Test and the Dunn to adjust for multiple comparison and adjust the *p*-value with Bonferroni’s correction).

**Table 16:** Contribution of dietary food sources to docosahexaenoic acid (DHA) intakes per age group among the ANIBES Study population.

Food Sources (g/Day)	Children	Adolescents	Adults	Elderly
Fish and shellfish	0.04 ^a^	0.03 ^a^	0.05 ^a,b^	0.07 ^b^
	(0.00–0.15)	(0.00–0.06)	(0.000–0.20)	(0.02–0.22)

Values are median (interquartile range) per group. Different superscript lowercase letters (^a^ and ^b^) indicate statistically significant difference between age groups (all differences are *p* ≤ 0.05; Kruskal–Wallis Test and the Dunn to adjust for multiple comparison and adjust the *p*-value with Bonferroni’s correction).

**Table 17:** Contribution of dietary food sources to omega-6 fatty acid intakes among the ANIBES Study population.

Food Sources (g/Day)	Omega-6 Fatty Acids (ω6)	Linoleic Acid (LA) (C18: 2*n*-6)	Arachidonic Acid (AA) (C20: 4*n*-6)
Oils and fats	3.32	3.32	0.00
	(1.87–5.83)	(1.87–5.82)	(0.00–0.00)
Meat and meat products	1.63	1.58	0.05
	(0.92–2.60)	(0.89–2.51)	(0.02–0.09)
Cereals and derivatives	0.99	0.99	0.00
	(0.62–2.03)	(0.61–2.01)	(0.00–0.00)
Fish and shellfish	0.07	0.03	0.00
	(0.00–1.43)	(0.00–1.41)	(0.00–0.02)
Eggs	0.27	0.27	0.00
	(0.05–0.54)	(0.05–0.54)	(0.00–0.00)
Milk and dairy products	0.18	0.17	0.00
	(0.10–0.28)	(0.10–0.26)	(0.00–0.00)
Vegetables	0.04	0.04	0.00
	(0.00–0.07)	(0.01–0.07)	(0.00–0.00)
Ready-to-eat meals	0.09	0.09	0.00
	(0.00–0.77)	(0.00–0.77)	(0.00–0.00)
Sauces and condiments	0.30	0.30	0.00
	(0.00–0.76)	(0.00–0.76)	(0.00–0.00)
Fruits	0.00	0.00	0.00
	(0.00–0.01)	(0.00–0.02)	(0.00–0.00)
Legumes	0.00	0.00	0.00
	(0.00–0.00)	(0.00–0.00)	(0.00–0.00)
Appetizers	0.00	0.00	0.00
	(0.00–0.08)	(0.00–0.08)	(0.00–0.00)
Sugars and sweets	0.00	0.00	0.00
	(0.00–0.07)	(0.00–0.06)	(0.00–0.00)
Supplements and meal replacement	0.00	0.00	0.00
	(0.00–0.00)	(0.00–0.00)	(0.00–0.00)
Non-alcoholic beverages	0.00	0.00	0.00
	(0.00–0.00)	(0.00–0.00)	(0.00–0.00)
Alcoholic beverages	0.00	0.00	0.00
	(0.00–0.00)	(0.00–0.00)	(0.00–0.00)

Values are median (interquartile range) per group.

**Table 18:** Contribution of dietary food sources to total omega-6 intakes per age group among the ANIBES Study population.

Food Sources (g/Day)	Children	Adolescents	Adults	Elderly
Oils and fats	4.02 ^a^	3.45 ^a,b^	3.39 ^a,b^	2.92 ^b^
	(2.16–6.39)	(1.86–5.87)	(1.89–5.83)	(1.68–4.92)
Meat and meat products	1.77 ^a^	2.01 ^b^	1.63 ^a^	1.29 ^c^
	(1.22–2.64)	(1.44–2.87)	(0.92–2.65)	(0.71–2.16)
Cereals and derivates	1.23 ^a^	1.37 ^a^	0.99 ^b^	0.77 ^b^
	(0.83–2.50)	(0.76–2.99)	(0.62–2.02)	(0.51–1.69)
Ready-to-eat meals	0.20 ^a^	0.17 ^a^	0.08 ^b^	0.06 ^b^
	(0.00–1.03)	(0.00–0.26)	(0.00–0.73)	(0.00–0.39)
Milk and dairy products	0.24 ^a^	0.21 ^a^	0.18 ^b^	0.16 ^b^
	(0.16–0.36)	(0.12–0.33)	(0.10–0.28)	(0.08–0.24)
Sauces and condiments	0.37 ^a^	0.42 ^a^	0.37 ^a^	0.00 ^b^
	(0.00–0.77)	(0.00–0.76)	(0.00–0.66)	(0.00–0.49)
Eggs	0.30 ^a^	0.27 ^a,b^	0.28 ^b^	0.32 ^b^
	(0.06–0.56)	(0.04–0.51)	(0.04–0.54)	(0.10–0.56)

Values are median (interquartile range) per group. Different superscript lowercase letters (^a^, ^b^ and ^c^) indicate statistically significant difference between age groups (all differences are *p* ≤ 0.05; Kruskal–Wallis Test and the Dunn to adjust for multiple comparison and adjust the *p*-value with Bonferroni’s correction).

**Table 19:** Contribution of dietary food sources to linoleic acid (LA) intakes per age group among the ANIBES Study population.

Food Sources (g/Day)	Children	Adolescents	Adults	Elderly
Oils and fats	4.01 ^a^	3.45 ^a,b^	3.39 ^a,b^	2.91 ^b^
	(2.16–6.39)	(1.86–5.87)	(1.89–5.83)	(1.68–4.91)
Meat and meat products	1.74 ^a,b^	1.97 ^a^	1.59 ^b^	1.23 ^c^
	(1.18–2.56)	(1.38–2.78)	(0.89–2.56)	(0.69–2.06)
Cereals and derivates	1.23 ^a^	1.37 ^a^	0.99 ^b^	0.77 ^c^
	(0.83–2.50)	(0.76–2.99)	(0.62–2.02)	(0.51–1.69)
Ready-to-eat meals	0.20 ^a^	0.16 ^a^	0.08 ^b^	0.06 ^b^
	(0.00–1.03)	(0.00–1.26)	(0.00–0.73)	(0.00–0.39)
Sauces and condiments	0.37 ^a^	0.42 ^a^	0.37 _a_	0.00 ^b^
	(0.00–0.77)	(0.00–0.76)	(0.00–0.76)	(0.00–0.49)
Eggs	0.30	0.27	0.28	0.32
	(0.06–0.58)	(0.04–0.54)	(0.04–0.54)	(0.10–0.56)
Milk and dairy products	0.23 ^a^	0.20 ^a^	0.17 _b_	0.15 ^b^
	(0.15–0.34)	(0.12–0.31)	(0.10–0.26)	(0.08–0.23)

Values are median (interquartile range) per group. Different superscript lowercase letters (^a^, ^b^ and ^c^) indicate statistically significant difference between age groups (all differences are *p* ≤ 0.05; Kruskal–Wallis Test and the Dunn to adjust for multiple comparison and adjust the *p*-value with Bonferroni’s correction).

**Table 20:** Contribution of dietary food sources to arachidonic acid (AA) intakes per age group among the ANIBES Study population.

Food Sources (g/Day)	Children	Adolescents	Adults	Elderly
Meat and meat products	0.053 ^a,b^	0.067 ^a^	0.050 ^b^	0.044 ^c^
	(0.034–0.082)	(0.034–0.093)	(0.024–0.087)	(0.016–0.077)
Milk and dairy products	0.006 ^a^	0.005 ^a,b^	0.004 ^b^	0.004 ^b^
	(0.003–0.009)	(0.003–0.009)	(0.002–0.007)	(0.002–0.007)
Fish and shellfish	0.001	0.001	0.001	0.002
	(0.000–0.019)	(0.000– 0.014)	(0.000–0.015)	(0.000–0.025)

Values are median (interquartile range) per group. Different superscript lowercase letters (^a^, ^b^ and ^c^) indicate statistically significant difference between age groups (all differences are *p* ≤ 0.05; Kruskal–Wallis Test and the Dunn to adjust for multiple comparison and adjust the *p*-value with Bonferroni’s correction).

## Data Availability

The data presented in this study are available on request from the corresponding author.
